# Depression in patients with anorectal fistulas and anal fissures: a propensity score-matched cohort study

**DOI:** 10.1007/s11136-024-03863-1

**Published:** 2024-12-14

**Authors:** Andreas Krieg, Ernst W. Kolbe, Michael Kaspari, Sarah Krieg, Sven H. Loosen, Christoph Roderburg, Karel Kostev

**Affiliations:** 1 Department of General and Visceral Surgery, Thoracic Surgery and Proctology, Medical Campus OWL, University Hospital Herford, Ruhr University Bochum, Schwarzenmoorstr. 70, 32049 Herford, Germany; 2 Department of Inclusive Medicine, University Hospital Ostwestfalen-Lippe, Bielefeld University, 33617 Bielefeld, Germany; 3 Department of Gastroenterology, Hepatology and Infectious Diseases, Medical Faculty of Heinrich Heine University Duesseldorf, University Hospital Duesseldorf, 40225 Duesseldorf, Germany; 4 IQVIA, 60549 Frankfurt, Germany

**Keywords:** Anal fissure, Anorectal fistula, Depression, Psychosocial impact, Quality of life, Chronic pain

## Abstract

**Purpose:**

Anal fissures and anorectal fistulas are diseases often associated with significant pain and prolonged discomfort, resulting in a significantly reduced quality of life (QoL). They are not only a surgical problem but also have a profound psychosocial impact and influence on QoL. The aim of this study was to investigate the incidence of depression in patients with these pathologies and to highlight the need to address the psychosocial aspects of these diseases.

**Methods:**

Data from the Disease Analyzer database of approximately 3,000 general practitioners in Germany were retrospectively analyzed. The retrospective cohort study included patients aged ≥ 18 years diagnosed with anal fissure (n = 15,467) or anorectal fistula (n = 3,520) between January 2005 and December 2022 and propensity score matched individuals without these disorders (n = 94,935). The primary outcome was a diagnosis of depression within five years of the index date. Kaplan–Meier curves and Cox regression analyses were used to analyze the association between the two anorectal diseases and depression.

**Results:**

After 5 years of follow-up, 13.0% of patients with anal fissure and 12.3% of patients with anorectal fistula were diagnosed with depression, compared with 9.7–10.3% in the control group (p < 0.001). Cox regression analysis showed a significant association between both diseases and depression (anal fissure: HR: 1.31; 95% CI: 1.25–1.38; anorectal fistula: HR: 1.30; 95% CI: 1.17–1.44).

**Conclusion:**

The results suggest that anal fissures or anorectal fistulas are significantly associated with a subsequent depression. This highlights the importance of multidisciplinary management that addresses both physical and psychosocial aspects to improve patient outcomes.

## Plain English summary

Anal fissures and anorectal fistulas are not only painful conditions, they can also affect a person's mental health and reduce their quality of life. The study investigated whether people with these conditions are more likely to develop depression, a mental health problem that can worsen their overall well-being. Therefore, the key question this research addresses is whether patients with anal fissures or anorectal fistulas are at higher risk of developing depression compared to those without these conditions. Main finding of this study is that people with anal fissures or anorectal fistulas are more likely to be diagnosed with depression within five years of their condition compared to people without these problems. The results show a clear link between these painful conditions and an increased risk of depression. This means that treating both the physical and mental health aspects of these conditions may be important in improving patients' quality of life

## Introduction

Anal fissures and anorectal fistulas are typical anorectal conditions that are frequently accompanied by considerable pain and discomfort.

The prevalence of anorectal fistulas in Europe is 1.69 per 10,000 population, with an annual incidence of 1.15 per 10,000 population [1]. Men in the third to fifth decade of life are affected three times more frequently than women [1, 2]. The pathogenesis of anorectal fistulas is complex and multifactorial. They often develop as a result of infection of the proctodeal glands, which may lead to a cyptoglandular abscess [3]. Approximately 37–66% of patients with an abscess eventually develop a fistula, which is an abnormal tubular connection between the anal canal and the skin or other organs [4, 5]. Infectious (e.g. prostatitis, tuberculosis), traumatic (iatrogenic injuries) or malignant (e.g. anal carcinoma) causes are less common. Anorectal fistulas play a special role in chronic inflammatory bowel disease, occurring in approximately 15–30% of patients with Crohn’s disease [6]. Based on a case–control study published by Wang et al., risk factors for anorectal fistula are body mass index > 25.0 kg/m^2^, high daily salt intake, history of diabetes, hyperlipidemia, dermatosis, anorectal surgery, history of smoking and alcohol consumption, sedentary lifestyle, excessive consumption of spicy/fatty foods, very infrequent exercise, and prolonged sitting on the toilet for defecation [7]. Typical symptoms include itching, discharge, discomfort, and possibly pain during defecation. However, anorectal fistulas can also develop from deepening of an anal fissure [8].

Anal fissures are longitudinal mucosal tears in the area of the highly sensitive anoderm, which typically begin in the posterior midline at the anocutaneous junction and can extend to the dentate line. The annual incidence of the disease is 11 per 10,000 individuals [9]. This disease affects men and women equally frequently, with the age peak in women occurring in the second to third decade of life and in men in the sixth decade of life [9]. The precise pathogenesis of anal fissures remains unclear. However, it is widely assumed that the majority of fissures are caused by direct trauma during the passage of hard stool or diarrhea [10, 11]. Furthermore, approximately 15% of women develop an anal fissure following childbirth [12]. In contrast, multiple fissures are primarily observed in an atypical lateral position in the context of Crohn’s disease, ulcerative colitis, HIV infection, neoplasia, syphilis, and tuberculosis [13]. In a case–control study published by Mapel and colleagues, an association was observed between anal fissure and concomitant diseases, including hypothyroidism, obesity, non-metastatic solid tumors, and weight loss [9]. Anal fissures are characterized by acute onset and are associated with severe pain and bleeding during attempts to defecate. While they typically resolve without intervention, chronic anal fissures may develop in cases where healing is incomplete. In such instances, the internal anal sphincter’s hypertonia is postulated to play a pathogenic role, potentially leading to reduced blood flow and delayed healing [10, 14, 15].

While cryptoglandular anal fistulas are surgically treated, the successful treatment of perianal fistulas associated with Crohn’s disease necessitates a multidisciplinary approach that incorporates anti-inflammatory therapy, which may be combined with surgery.

Treatment of acute anal fissures includes a diet rich in fiber, local application of calcium antagonists or nitrates, such as glyceryl trinitrate, as well as the injection of botulinum toxin into the internal anal sphincter or the use of anal dilators. In the treatment of chronic anal fissure, surgical procedures such as fissurectomy (with or without botulinum toxin injection), also in combination with an advancement flap, or lateral internal sphincterotomy, are used.

In any case, anal fissures and anorectal fistulas are associated with a number of accompanying symptoms, ranging from considerable pain to discomfort, fear of defecation, and psychosocial distress, which can significantly impact the quality of life (QoL) of those affected [16, 17]. In the case of anal fissures, it appears that pain plays a significant role, as evidenced by the findings of the study conducted by Griffin and colleagues [16]. The QoL was found to be markedly impaired in comparison to the general population, and the pain associated with the anal fissure appeared to impact all aspects of psychological and physical well-being [16]. Interestingly, the duration of the fissure seemed to have no discernible influence on physical and mental health. The study by Owen et al. revealed a tendency towards impaired QoL in patients with anal fistulas in comparison to the general population. Moreover, patients with recurrent fistulas exhibited a poorer QoL than those with primary fistulas. In anorectal fistulas, however, faecal incontinence plays a significant role in the impairment of QoL, in addition to the pain. It is well established that faecal incontinence has a negative impact on QoL [18]. In particular, in cases of transphincteric fistulas, where the sphincter muscle is divided, faecal incontinence represents a postoperative risk, with the potential to significantly impair the patient’s QoL [19–21]. However, the overall incidence of incontinence varies by up to 45%, depending on the type of fistula and surgical procedure [20]. Studies have demonstrated that even in cases of simple fistulas, there is a risk of incontinence, albeit at a lower rate than in cases of complex fistulas [22]. However, it is noteworthy that a study published by Cioli and colleagues indicates that an altered emotional state may also be a significant factor in the development of anal fistulas [23]. This underscores the importance of psychological screening in patients with anorectal disorders. While a correlation between QoL and depression may appear plausible at first glance, to the best of our knowledge, no studies have specifically investigated whether an anorectal fistula is associated with the onset of depression. In contrast, studies on chronic anal fissures, albeit on very small patient populations, have provided initial evidence that these patients complain of psychiatric symptoms such as depression [24–26].

Considering that QoL and depression are closely related [27], our aim was to investigate the cumulative incidence of depression in patients with anal fissures or anorectal fistulas compared to a population without anal fissures or fistulas in a large German cohort.

## Methods

### Database

This retrospective cohort study utilized data from the Disease Analyzer database (IQVIA). This database, which has been employed in several previous studies on depression [28, 29], contains anonymized data on diagnoses, prescriptions, as well as basic medical and demographic information from computer systems used in office-based practices [30]. The database encompasses approximately 3,000 office-based practices in Germany. The sampling method for the Disease Analyzer database uses statistics from the German Medical Association to design the panel according to specialist group, German federal state, community size category, and age of the physician. It has been demonstrated that the panel of practices included in the Disease Analyzer database is representative of general and specialized practices in Germany [30].

### Study population

**Fig. 1:**
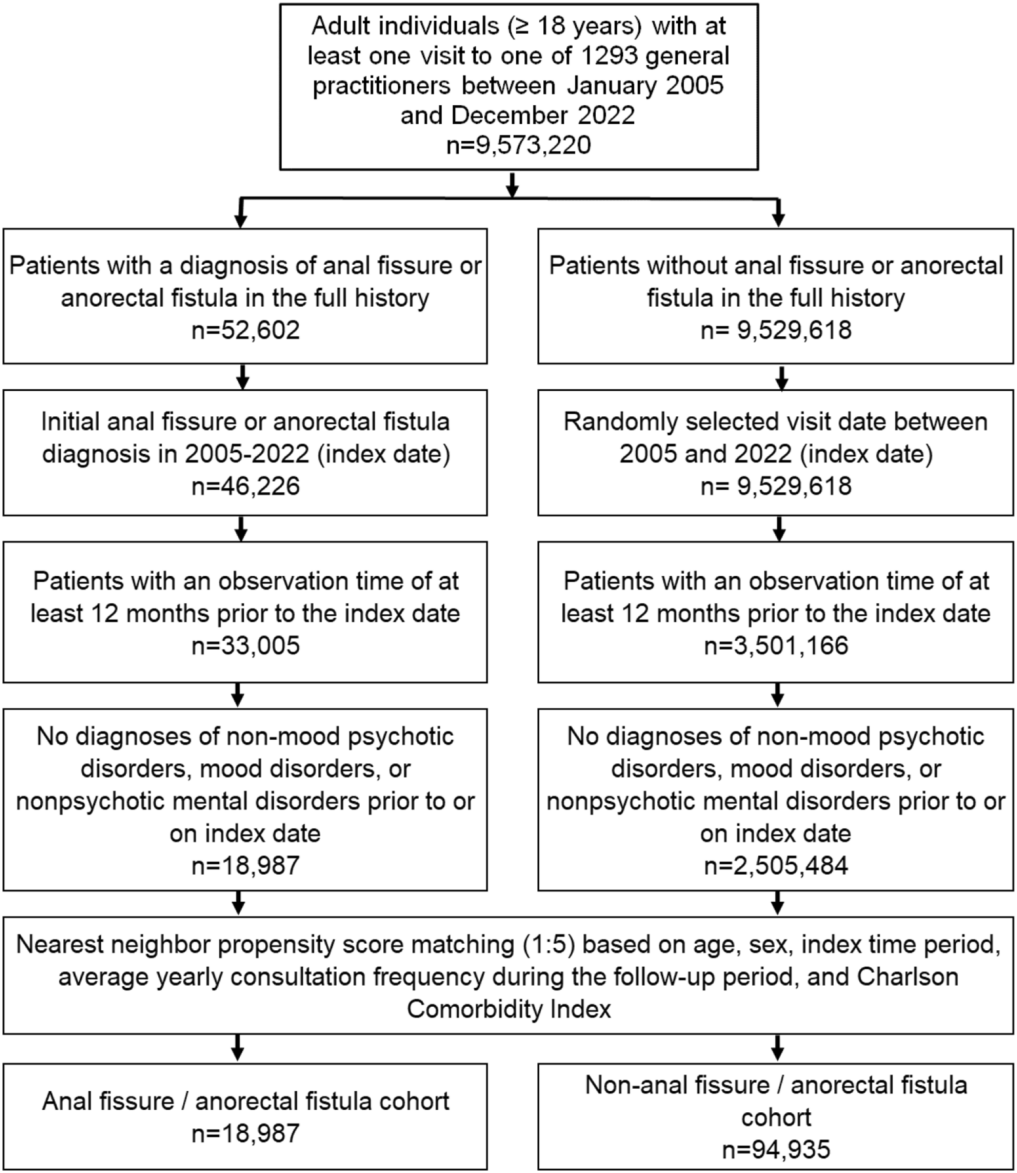
Selection of study patients

The International Statistical Classification of Diseases and Related Health Problems, 10th Revision (ICD-10 of WHO) was used as the official classification of diagnoses to search the database for the following conditions: anal fissure, anarectal fistula and depression. Accordingly, patients aged ≥ 18 years with an initial diagnosis of anal fissure (ICD-10: K60.0, K60.1, K60.2) or anorectal fistula (ICD-10: K60.3, K60.4, K60.5) in 1,284 general practices in Germany between January 2005 and December 2022 (index date; Fig. 1) were included in our study. Diagnoses documented by general practitioners (GPs) may also have been previously diagnosed by specialists in other practices or in hospitals. It should be noted that the diagnostic process and method used to diagnose depression in our cohort are not documented in our database. Accordingly, we were similarly unable to provide details regarding the diagnostic tools employed and the specialists responsible for diagnosing depression. In Germany, however, GPs frequently document diagnoses made by other specialists, such as psychiatrists. First documented anal fissure and anorectal fistula diagnosis by GPs between January 2005 and December 2022 was considered index date. An additional inclusion criterion was an observation time of at least 12 months prior to the index date. Patients with non-mood psychotic disorders (ICD-10: F20-F29), mood disorders (ICD-10: F30-39), or non-psychotic mental disorders (ICD-10: F40-48) prior to or at index date were excluded.

After applying similar inclusion criteria, individuals without anal fissure or fistula diagnoses were matched to patients with these diseases using nearest neighbor propensity score matching (5:1) based on age, sex, index time period, average yearly consultation frequency during the follow-up, and Charlson Comorbidity Index (CCI). The CCI includes a wide range of comorbidities including diabetes, cancer, cardiovascular pulmonary, gastrointestinal, liver, renal diseases, and others [31]. For the non- anal fissure and fistula cohort, the index date was that of a randomly selected visit between January 2005 and December 2022 (Fig. 1). A standardized mean difference (SMD) of less than 0.1 was allowed indicating that adequate covariate balance between cohorts has been achieved.

### Study outcomes and statistical analyses

The primary outcomes of this study were the initial diagnoses of depression (ICD-10: F32, F33) within five years following the index date, analyzed in relation to anal fissure and anorectal fistula. The five-year cumulative incidence of depression in cohorts with and without anal fissure and anorectal fistula was examined using Kaplan–Meier curves, with comparisons made via the log-rank test. Additionally, a univariable Cox regression analysis was performed to evaluate the association between anal fissure, anorectal fistula, and depression. The results of the Cox regression model are presented as hazard ratios (HRs) with 95% confidence intervals (CIs). Separate Cox regression analyses were also conducted for men, women, and six distinct age groups. Given the multiple comparisons, a p-value of < 0.01 was deemed statistically significant. All analyses were executed using SAS version 9.4 (SAS Institute, Cary, NC, USA).

In the present study, the variables employed were limited to age, sex, and diagnoses. Age was not missing, as only patients with documented age were included in the initial selection of adult patients. A small proportion of patients in the database (less than 1%) had missing age data. Thus, these patients were excluded from the beginning. Information pertaining to the patients’ sexes was available for the entirety of the patient population. In regard to diagnoses, it should be noted that the data in question pertain to electronic medical records (EMR), which may or may not include information related to specific diseases or conditions. In other words, a patient may or may not have been diagnosed with a particular disease or condition. Therefore, no data are missing in this case with regard to diagnoses. It is possible that some diseases may have been underdiagnosed by physicians, but we do not have the necessary information to confirm this. We only have data on whether a diagnosis is available or not. Dealing with missing data is a necessary and possible approach, as it allows for the inclusion of cases where patients do not respond to questions, for example, when people complete surveys. However, as our study is based on EMR, we did not conduct further analysis on missing data.

## Results

### Basic characteristics of the study sample

This study encompassed 18,987 individuals diagnosed with either anal fissure (n = 15,467) or anorectal fistula (n = 3,520), alongside 94,935 individuals without these conditions. Table 1 presents the main characteristics of the study participants. The mean age was 48.2 years (standard deviation (SD): 17.5 years), with 38.3% being women. On average, patients visited their GPs 6.4 times annually during the follow-up period. The matched pairs design ensured no significant differences between the two cohorts regarding age, sex, visit frequency, and CCI (Table 1).

**Table 1:** Baseline characteristics of the study sample (after propensity score matching)

Variable	Proportion among individuals with anal fissure and anorectal fistula (%) n = 18,987	Proportion among individuals without anal fissure and anorectal fistula (%) n = 94,935	SMD
Age (Mean, SD)	48.2 (17.5)	48.2 (17.5)	0.011
Age ≤ 30	3,600 (19.0)	17,997 (19.0)	
Age 31–40	3,621 (17.2)	16,298 (17.2)	
Age 41–50	3,787 (19.9)	18,901 (19.9)	
Age 51–60	3,557 (18.7)	17,860 (18.8)	
Age 61–70	2,380 (12.5)	11,918 (12.5)	
Age > 70	2,402 (12.7)	11,961 (12.6)	
Women	7,279 (38.3)	36,405 (38.3)	0.000
Men	11,708 (61.7)	58.530 (61.7)	
Number of physician visits per year during the follow-up (Mean, SD)	6.4 (4.2)	6.4 (4.2)	-0.005
Charlson Comorbidity Score (Median, IQR)	1 (2)	1 (2)	0.004
CCI 0	7,269 (38.3)	36,358 (38.3)	
CCI 1	4,973 (26.2)	25,112 (26.4)	
CCI 2	2,713 (14.3)	13,445 (14.2)	
CCI ≥ 3	4,032 (21.2)	20.020 (21.1)	

Proportions of patients in % given, unless otherwise indicated. *SD* standard deviation, *IQR* interquartile range

### Association of anal fissure and anorectal fistula with subsequent depression diagnosis

**Fig. 2:**
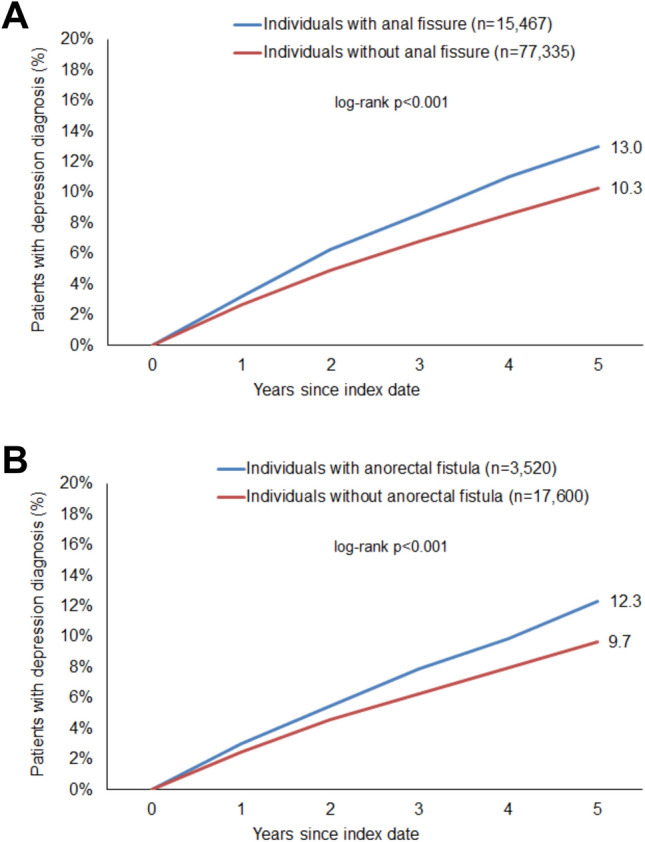
Cumulative incidence of depression in individuals with and without anal fissure (**A**) and anorectal fistula (**B**)

After up to five years of follow-up, 13.0% of patients with anal fissure and 12.3% of patients with anorectal fistula were diagnosed with depression, compared to 9.7–10.3% of the matched healthy cohort (p < 0.001) (Fig. 2). Regression analysis revealed a significant association between anal fissure and subsequent depression diagnosis (HR: 1.31; 95% CI: 1.25–1.38), as well as between anorectal fistula and subsequent depression diagnosis (HR: 1.30; 95% CI: 1.17–1.44) (Table 2). These associations were consistent across both women and men. Age-stratified analysis indicated that the association between anal fissure and depression was not significant in the ≤ 30 age group, with the strongest association observed in the 61–70 age group. Similarly, the association between anorectal fistula and depression was not significant in the ≤ 30, 31–40, 41–50, and 51–60 age groups, but was very strong in the 61–70 and > 70 age groups (Table 2).

**Table 2:** Association between anal fissure and anorectal fistula and subsequent depression in patients followed in general practices in Germany (univariable Cox regression models)

	Anal fissure		Anorectal fistula	
Sub-cohorts	HR (95% CI)	P value	HR (95% CI)	P value
Total	1.31 (1.25–1.38)	< 0.001	1.30 (1.17–1.44)	< 0.001
Age ≤ 30	1.17 (1.03–1.32)	0.013	1.14 (0.86–1.50)	0.368
Age 31–40	1.22 (1.09–1.38)	< 0.001	1.17 (0.91–1.49)	0.224
Age 41–50	1.41 (1.27–1.55)	< 0.001	1.29 (1.05–1.59)	0.017
Age 51–60	1.32 (1.19–1.47)	< 0.001	1.16 (0.92–1.47)	0.209
Age 61–70	1.46 (1.27–1.68)	< 0.001	1.86 (1.38–2.50)	< 0.001
Age > 70	1.30 (1.13–1.49)	< 0.001	1.60 (1.13–2.27)	0.008
Women	1.32 (1.23–1.42)	< 0.001	1.29 (1.07–1.57)	0.009
Men	1.30 (1.22–1.39)	< 0.001	1.30 (1.15–1.47)	< 0.001

## Discussion

The results of our study indicate a significant association between anal fissures or anorectal fistulas and subsequent depression. In particular, our findings suggest that patients diagnosed with anal fissures or anorectal fistulas exhibit an approximately 30% increased incidence of developing depression within five years of diagnosis, when compared to a matched cohort without these conditions. While a considerable number of studies have focused on the local treatment of both diseases, only a limited body of research has examined the impact of the disease course on the mental health of patients.

There is evidence to suggest that anal fistulas have a significant impact on the QoL of those affected [17, 32]. A recently published study, which employed a qualitative methodology utilizing semi-structured patient interviews, revealed that the psychological impact of the fistula was pervasive [32]. In consequence, the stress of coping with the symptoms gave rise to a state of constant anxiety, with patients experiencing worry in anticipation of the symptoms. For example, patients reported that managing their symptoms on a daily basis, ensuring they always had additional bandages or equipment with them, and wearing secure clothing was stressful. Additionally, the interviews revealed that being constantly reminded of their illness and its impact on their intimacy was distressing and exhausting for those affected.

Furthermore, faecal incontinence has a considerable impact on QoL in anorectal fistulas. This is an unsurprising finding, given that previous research has demonstrated a negative impact of faecal incontinence on QoL [18]. The functionality of the sphincter and the subsequent impact on faecal incontinence are largely influenced by the extent of the fistula and the nature and extent of surgical intervention [19–21]. The study by Bokhari et al. demonstrated that 23% of patients experienced new or worsening incontinence following surgical fistula repair, with only 6% reporting severe faecal incontinence [22]. Anorectal fistulas are classified as either simple or complex, depending on the trajectory of the fistula tract through the sphincter muscle, the number of fistula openings, and the presence of abscesses. Notably, patients with complex fistulas who underwent sphincter splitting exhibited a markedly higher prevalence of severe incontinence (13%) compared to those in whom the sphincter was preserved (0%) [22].

To date, several studies have focused on the development of disease-specific tools to measure QoL in patients with anorectal fistula. Iqbal et al. [33] developed the Anal Fistula QoL Scale (AF-QoL), a 22-item instrument with good validity. The Crohns Anal Fistula QoL Scale (CAF-QoL) was developed by Adegbola et al. [34]. This questionnaire has 26 items. In addition, chronic anal fistula has a significant impact on patients’ QoL and negatively affects their physical and mental health [16]. A recently published case–control study by Navarro-Sánchez et al. included 35 patients with anal fissures and 32 controls [25]. The study demonstrated higher scores on the Hospital Anxiety and Depression Scale and lower scores on the SF-12 Health Survey and the Multicultural QoL Index. Thus, these findings suggest a higher incidence of depression and poorer QoL in patients with chronic anal fissures.

Prior research has also documented an association between other painful conditions of the anal region and mental health. While functional anorectal pain was associated with a 50% prevalence of depression in the study by Dong and colleagues [35], we recently demonstrated an association between hemorrhoids and depression in a matched cohort [36].

However, the majority of studies examining the relationship between QoL, psychological stress, and anorectal fistulas or anal fissures have been conducted with relatively small patient populations. Additionally, these studies have not specifically addressed the incidence of depression among individuals affected by these diseases. Thus, it is important to be aware of the high incidence of depression in these patients in comparison to those without the condition, as this knowledge is relevant for the adequate treatment and care of these patients.

A robust relationsship between depression and QoL is well documented [27]. However, this relationship appears to be bidirectional, with poor QoL potentially leading to depression and vice versa [37]. It is noteworthy that the QoL improves with the resolution of depressive symptoms, though it may not reach the level of those without depression [37]. From our perspective, it is reasonable to conclude that the psychosocial stress associated with anal fissures and anorectal fistulas, such as feelings of shame and social isolation, contribute to the development of depression. Our findings, therefore, highlight the necessity to integrate both the treatment of depression and the improvement of QoL into the treatment of anorectal fistulas and anal fissures.

It can be hypothesized that there is a potential link between depression and pain due to anal fissures and anorectal fistulas, which is consistent with the data presented. Both diseases have common mechanisms involving neurotransmitters, neuromodulators, and neurohormones [38]. The co-occurrence and mutual reinforcement of chronic pain and depression are associated with overlapping changes in neuroplasticity [39]. Key neurobiological factors involved in both disorders include alterations in serotonin levels, proinflammatory cytokines, and brain neurotrophic factor [40]. Nevertheless, the relationship between pain and depression is also bidirectional, with chronic pain increasing the likelihood of developing depression and vice versa [40].

Furthermore, this study revealed a more pronounced association between anal fissures, anorectal fistulas, and depression in older patients. One potential explanation for this phenomenon is that older individuals tend to experience a higher prevalence of chronic comorbidities. As demonstrated by Makovski and colleagues [41] in their meta-analysis, an increase in the number of comorbidities is significantly associated with a reduction in QoL, which, in turn, exerts an influence on the development of depression. Furthermore, the often chronic and protracted course of treatment for anal fissures and anorectal fistulas, which is associated with repeated visits to the healthcare provider, can result in not only additional physical limitations but also greater dependence on others due to potential mobility restrictions and an increased sense of loss of meaning. These factors can render older individuals more susceptible to depression or exacerbate existing depressive symptoms.

The present study has several notable strengths, including the use of a relatively large and representative cohort and the use of propensity score matching to control for potential confounding factors. Moreover, the analysis of data from the Disease Analyzer database allows for the examination of a diverse and heterogeneous patient cohort, thereby enhancing the generalizability of the findings.

Additionally, it is necessary to acknowledge the limitations of this study. Primarily, this is a retrospective observational study, which makes it impossible to establish causal relationships. Secondly, the diagnoses are based on medical records, which may be subject to misclassification or underreporting. Thirdly, potentially relevant factors such as the severity of the anal fissure or anorectal fistula and therapeutic modalities were not taken into account, which could influence the results. Furthermore, we had no information regarding the methodology employed to diagnose the patient’s depression. Specifically, we had no knowledge of the diagnostic tool utilized, whether a questionnaire or another instrument, or the expertise of the diagnosing physician, whether a general practitioner or a psychiatrist. Additionally, we had no data from hospitals or other specialists for the same patient. Consequently, we could not ascertain the patient’s medical history or treatment plans at other medical facilities.

Thus, to further investigate the causal relationship between anal fissures, anorectal fistulas, and depression, it is necessary to conduct high-level evidence studies with a prospective, randomized design. In addition, qualitative studies would be beneficial to gain a deeper understanding of the psychosocial factors and needs of this patient population. Moreover, intervention studies may contribute to the development of effective strategies for the prevention and treatment of depression in patients with anal fissures and anorectal fistulas.

## Conclusion

In conclusion, our study underscores the necessity of recognizing anal fissures and anorectal fistulas not only as physical but also as mental health concerns. In the context of treating anorectal fistulas and anal fissures, healthcare providers should consider the possibility of co-existing depression and integrate appropriate screening and treatment modalities into their practice. The removal of taboos surrounding these conditions and the encouragement of an open approach can facilitate a reduction in the psychosocial burden on those affected.

## Data Availability

The data that support the findings of this study are available on request from the corresponding author on reasonable request.
